# Two new species of the genus *Panorpa* (Mecoptera, Panorpidae) from eastern China and a new synonym

**DOI:** 10.3897/zookeys.874.36314

**Published:** 2019-09-09

**Authors:** Ji-Shen Wang, Xiao-Tong Gao, Bao-Zhen Hua

**Affiliations:** 1 Key Laboratory of Plant Protection Resources and Pest Management, Ministry of Education, Entomological Museum, College of Plant Protection, Northwest A&F University, Yangling, Shaanxi 712100, China Northwest A&F University Yangling China

**Keywords:** Anhui, biodiversity, Oriental region, scorpionfly, taxonomy, Zhejiang

## Abstract

*Panorpa* Linnaeus, 1758 is the largest genus in the scorpionfly family Panorpidae. Herein we describe two new species from eastern China, *Panorpa jinhuaensis***sp. nov.** from Jinhua, Zhejiang Province and *Panorpa menqiuleii***sp. nov.** from Yuexi and Huoshan, Anhui Province. *Panorpa wrightae* Cheng, 1957 from Mount Mogan, Zhejiang Province is considered to be a junior subjective synonym of *Panorpa mokansana* Cheng, 1957 from the same locality. *Panorpa mokansana* Cheng, 1957 is redescribed and illustrated in detail. A key to species of *Panorpa* from eastern China is also provided.

## Introduction

Panorpidae is the largest family in the order Mecoptera (Bicha 2018; Lin et al. 2019), with ca. 500 extant species in eight genera known hitherto (Wang and Hua 2017, 2019; Gao and Hua 2019; Hu et al. 2019). They are commonly called “scorpionflies” due to their enlarged and upcurving male genitalia, which superficially resemble the stinger of scorpions (Dunford and Somma 2008; Byers 2009). The adults usually scavenge dead invertebrates, and occasionally feed on vegetative materials such as nectar and pollen grains (Palmer 2010). They often inhabit high-elevated moist forests, and are typically observed sitting on the upper surfaces of leaves of herbs or shrubs (Byers and Thornhill 1983; Wang and Hua 2016, 2019; Bicha 2018).

The Holarctic *Panorpa* Linnaeus, 1758 is the largest genus (ca. 260 spp.) in Panorpidae, and has been regarded a paraphyletic group in several studies (Willmann 1989; Misof et al. 2000; Whiting 2002; Ma et al. 2012; Hu et al. 2015; Miao et al. 2019). This genus can be differentiated from the Oriental genera *Leptopanorpa* MacLachlan, 1875 and *Neopanorpa* van der Weele, 1909 mainly by the vein 1A ending at the same level or distal (cf. proximal) to the origin of Rs, and two (cf. one) cross-veins between 1A and 2A in forewings, with *Panorpa bashanicola* Hua, Tao & Hua, 2018 as an exception (Hua et al. 2018).

An unofficial rank, “species group”, is adopted in the taxonomy of *Panorpa* by many researchers (Esben-Petersen 1921; Issiki 1933; Carpenter 1938; Cheng 1957b). Nine species groups were proposed by Issiki (1933) for the East Asian *Panorpa*. In eastern China (including Anhui, Fujian, Jiangsu, Jiangxi, Shandong, and Zhejiang Provinces, as well as Shanghai City), 21 species of *Panorpa* belonging to three groups have been documented (Wang and Hua 2017). For example, *Panorpa baohwashana* Cheng, 1957 (Jiangsu) from the *P. amurensis* group, *Panorpa kellogi* Cheng, 1957 (Fujian) from the *P. japonica* group, and *Panorpa obliqua* Carpenter, 1945 (Jiangxi) and *Panorpa implicata* Cheng, 1957 (Fujian) from the *P. wormaldi* group.

In this paper, we illustrate and describe two new species of *Panorpa* from eastern China. They resemble *P. waongkehzengi* Navás, 1935 (Jiangxi) mainly by the non-elongated cylindrical male A6–A7 (abdominal segments VI‒VIII), and the twisted posterior arms in the female medigynium, but can be readily differentiated from the latter by the male genitalia. In addition, *Panorpa wrightae* Cheng, 1957 from Mount Mogan is considered to be a junior subjective synonym of *Panorpa mokansana* Cheng, 1957 from the same locality. A key to species of *Panorpa* from eastern China is also provided.

## Material and methods

**Figure 1.**
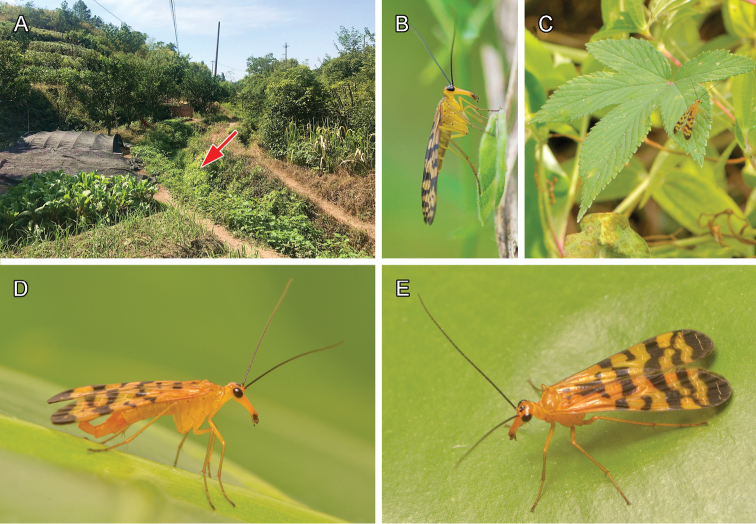
*Panorpa jinhuaensis* sp. nov. **A** habitat **B** female, lateral view, photo by En Lin **C** male staying on a leaf of *Humulus scandens***D** male, lateral view **E** female, dorso-lateral view. Red arrow in **A** points to an irrigation canal with dense herbaceous groundcover, where the specimens were caught. **A, C–E** taken on October 2, 2018, and **B** taken on April 19, 2018 from Jinhua City.

Adult scorpionflies were caught with collecting nets, and preserved in 95% ethanol or pinned as permanent collections. The specimens examined are deposited in the Entomological Museum, Northwest A&F University, Yangling (**NWAU**) and the Institute of Zoology, Chinese Academy of Sciences, Beijing (**IZAS**). Specimens were observed under a Nikon SMZ 1500 Stereoscopic Zoom microscope. Measurements of right wings were made with a vernier caliper. The lengths of wings were measured from the base to the apex, and widths from the ending of M_4_ to the costal margin vertically. Photographs were taken with a Nikon D7000 digital camera except Figure 1B with a Nikon D7100 digital camera. All pictures were further adjusted and assembled with Adobe Photoshop CS4.

Terminology follows Byers (1989), Hua et al. (2018) and Wang and Hua (2019). The following abbreviations and acronyms are applied: A1, first abdominal segment (and so forth for other segments); T1, first tergum (and so forth for other segments); FL, forewing length; FW, forewing width; HL, hindwing length; and HW, hindwing width.

## Taxonomy

### Panorpa jinhuaensis sp. nov.

Taxon classificationAnimaliaMecopteraPanorpidae

B15D3A94B996591E9F044D16A2371F34

http://zoobank.org/35AC533C-04E8-4FD1-B0C2-D43C43461695

Figures 1 , 2

#### Type material.

**Holotype**: ♂ (NWAU), CHINA: Zhejiang Province, Jinhua City [金华市], southern slope of Mount Jinhua [金华山], Zhizhe (Wise Man) Temple [智者寺] (29°10'03"N, 119°37'21"E, 104 m), 2.x.2018, leg. Ji-Shen Wang; **Paratypes**: 20♂13♀ (NWAU), same data as for the holotype.

#### Etymology.

The specific name refers to the type locality, Jinhua City.

#### Diagnosis.

This new species is superficially similar to *Panorpa waongkehzengi* Navás, 1935 from Jiangxi, but can be readily differentiated from the latter by: in males, 1) apex of epandrium broadly rounded (cf. abruptly narrowed); 2) inner margin of hypovalve straight (cf. with an inner process); 3) paramere long and exceeding apex of gonocoxites (cf. short and not exceeding apex of gonocoxites); 4) apical portion of paramere spiral (cf. straight); 5) parameres crossed subbasally (cf. not crossed); and in females, 6) main plate of medigynium moderately developed (cf. poorly developed).

#### Measurements.

Male FL 10.2‒10.8 mm, FW 2.6‒2.8 mm; HL 9.0‒9.5 mm, HW 2.4‒2.6 mm. Female FL 11.0‒11.8 mm, FW 3.0‒3.2 mm; HL 10.0‒10.6 mm, HW 2.8‒3.0 mm.

#### Description-male.

**Figure 2.**
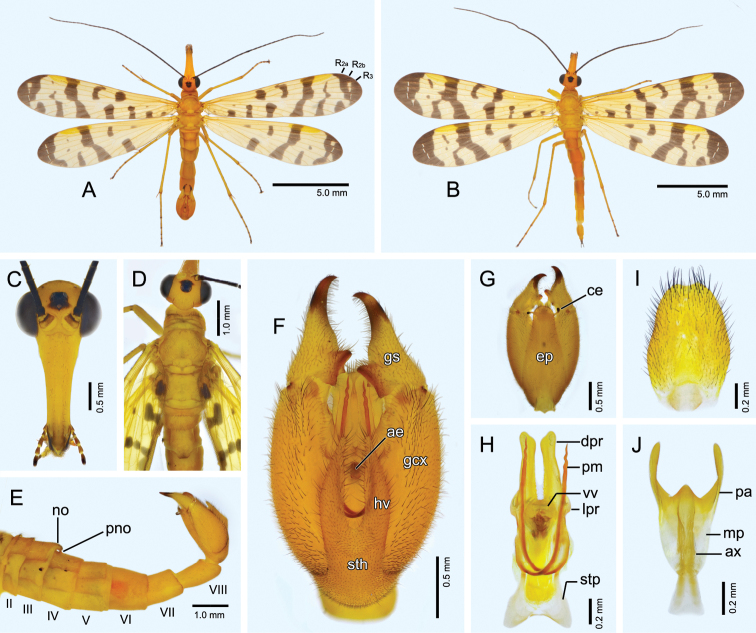
*Panorpa jinhuaensis* sp. nov. **A, C–H** male **B, I, J** female. **A, B** Habitus, dorsal view **C** head, frontal view **D** dorsum, dorsal view **E** abdomen, lateral view **F, G** genital bulb, ventral and dorsal views, respectively **H** aedeagal complex, ventral view **I** subgenital plate, ventral view **J** medigynium, ventral view. **ae**, aedeagus; **ax**, axis; **ce**, cercus; **dpr**, dorsal process; **ep**, epandrium; **gcx**, gonocoxite; **gs**, gonostylus; **hv**, hypovalve; **lpr**, lateral process; **mp**, main plate; **no**, notal organ; **pa**, posterior arm; **pm**, paramere; **pno**, postnotal organ; **sth**, stalk of hypandrium; **stp**, stalk of paramere; **vv**, ventral valve.

*Head* (Fig. 2C). Vertex, occiput and frons orange-yellow, with lateral margins of occiput slightly darkened. Black spot enclosing ocellar triangle and slightly spreading anteriorly. Compound eyes black, narrower than base of rostrum. Rostrum orange-yellow, stout, sparsely covered with short setae, with its length approximately 2.6 times as long as basal width. Labrum dark yellowish brown. Maxillary palp with basal four segments dark yellowish brown and distal segment black. Scape yellowish brown with distal margin dark brown; pedicel and flagellum black; flagellomeres 34‒36.

*Thorax* (Fig. 2D). Pronotum unevenly orange-yellow, with 10‒12 stout setae along anterior margin. Meso- and metanotum orange-yellow and sparsely covered with short setae; scutellar arms slightly deepened. Pleura and legs orange-yellow, with distal tarsomere blackish.

*Wings* (Fig. 1D, 2A). Membrane subtranslucent, strongly tinged with yellow and fading toward apex. Markings black. Veins yellowish brown except apical crossveins pale white. Pterostigma orange-yellow and distinct. Forewing apical band broad, usually with 1‒3 hyaline windows enclosing crossveins between R_3_ and M_1_, and a separated spot at ending of M_2_ posteriorly; apical branch of pterostigmal band variable: intact (Fig. 1D) or detached with pterostigmal band and greatly elongated anteriorly (Fig. 2A); basal branch of pterostigmal band intact and slightly broader than apical branch; marginal spot C-shaped; basal band split into two large spots; an additional transverse band extending from ending of 2A to CuA; basal spot shifted posteriorly and along anal margin; an additional small spot anterior to 3A; R_2_ bifurcated. Hindwing similar to forewings but bearing relatively reduced markings: basal band split into a large spot along posterior margin, and a small indistinct spot slightly distal to ORs; spots and band proximal to basal band absent.

*Abdomen* (Fig. 2A, D, E). Terga II‒V orange-yellow and slightly darkened at lateral margins, sparsely covered with black short setae; corresponding sterna lighter. Notal organ on posterior margin of T3 slightly prolonged posteriorly with truncated apex, bearing dense black setae on hind margin, and covering acute postnotal organ on anterior portion of T4. A6‒A8 orange-yellow, cylindrical. A6 as long as A5 and devoid of anal horns. A7 slightly shorter and narrower than A6. A8 nearly as long as A7, slightly enlarged posteriorly with a beveled apex.

Genital bulb (Fig. 2F, G) long oval, mostly orange-yellow except distal third of gonostyli blackish. Epandrium long and broad, evenly tapering toward rounded apex bearing dense long setae. Cerci clavate, orange-yellow in basal half and black in distal half. Hypandrium with short broad stalk and a pair of longer hypovalves; each hypovalve tapering toward apex, and bearing long stout setae along inner margin. Gonocoxites stout, approximately 1.6 times as long as gonostyli; gonostyli slightly curved on outer margin, and with a rounded median tooth and a large bowl-shaped basal process on inner margin. Paramere (Fig. 2H) slender, with greatly expanded stalk basally; connected to aedeagus through curved bridge-like process dorsally; and extending slightly beyond apex of gonocoxites with spiral and acute apex. Two parameres crossed basal to ventral aedeagal valves. Dorsal aedeagal processes greatly elongated posteriorly with slightly enlarged and beveled apex, and bearing a row of short setae along basal third of inner margin; lateral processes short and stout.

#### Description-female.

Similar to males except relatively denser wing markings. In fore- and hindwings, pterostigmal band with apical branch intact, scattered into 1‒3 small spots anteriorly (Figs 1B, 2B) or slender and extending to anterior margin, forming an H-shaped pattern (Fig. 1E).

*Female genitalia* (Fig. 2I, J). Subgenital plate oval, slightly tapering toward shallowly emarginate apex, and bearing long stout setae marginally. Medigynium with moderately developed main plate; posterior arms slightly shorter than main plate and twisted ventrally in distal half; axis longer than posterior arms, with apodemes extending beyond main plate and slightly divergent anteriorly; posterior apex of axis subtriangular and slightly extending beyond main plate.

#### Distribution.

China, Zhejiang (Jinhua).

#### Remarks.

The new species inhabits dense herbaceous ground cover aside an irrigation canal in a suburban field (Fig. 1A) with a surprisingly low elevation of 104 m (most *Panorpa* species in eastern China prefer higher mountainous regions above 600 m). The species is sympatric with another autumnal species, *Panorpa tetrazonia* Navás, 1935, which can be differentiated from the former by its larger body size (FL 12.0–13.0 mm) and brown body color. Apparently, *P. jinhuaensis* sp. nov. represents the dominant species at the locality, because only three males and two females of the latter were collected on the same day (2.x.2018). In addition, a female adult of *P. jinhuaensis* sp. nov. was photographed (Fig. 1B) in spring (19.iv.2018), likely indicating the bivoltinism of this species.

### Panorpa menqiuleii sp. nov.

Taxon classificationAnimaliaMecopteraPanorpidae

6247DEA9587654CB87B02663C25BF84F

http://zoobank.org/A479662E-1E4B-4BDD-9DCB-C57950F573C0

Figure 3

#### Type material.

**Holotype**: ♂ (NWAU), CHINA: Anhui Province, Yuexi County [岳西县], Yaoluoping [鹞落坪], 15.viii.2013, leg. Qiu-Lei Men; **Paratypes**: 1♂3♀ (NWAU), same data as for the holotype; 2♂5♀ (IZAS), Huoshan County [霍山县], Majiahe [马家河], 800 m, 31.viii.1978, leg. Wan-Cheng Fu.

#### Etymology.

The specific name is dedicated to the main collector of the type specimens, Qiu-Lei Men, for his generous help to our present research.

#### Diagnosis.

The new species is superficially similar to *Panorpa waongkehzengi* Navás, 1935 from Jiangxi and *P. jinhuaensis* sp. nov. in general appearance, but can be readily differentiated from the latter two by the presence (cf. absence) of a black pattern on the occiput, and a greatly shortened (cf. long) axis in the female medigynium.

#### Measurements.

Male FL 10.0‒10.2 mm, FW 2.8 mm; HL 9.0‒9.2 mm, HW 2.7 mm. Female FL 10.8‒11.0 mm, FW 2.9 mm; HL 9.5‒10.0 mm, HW 2.8 mm.

#### Description-male.

**Figure 3.**
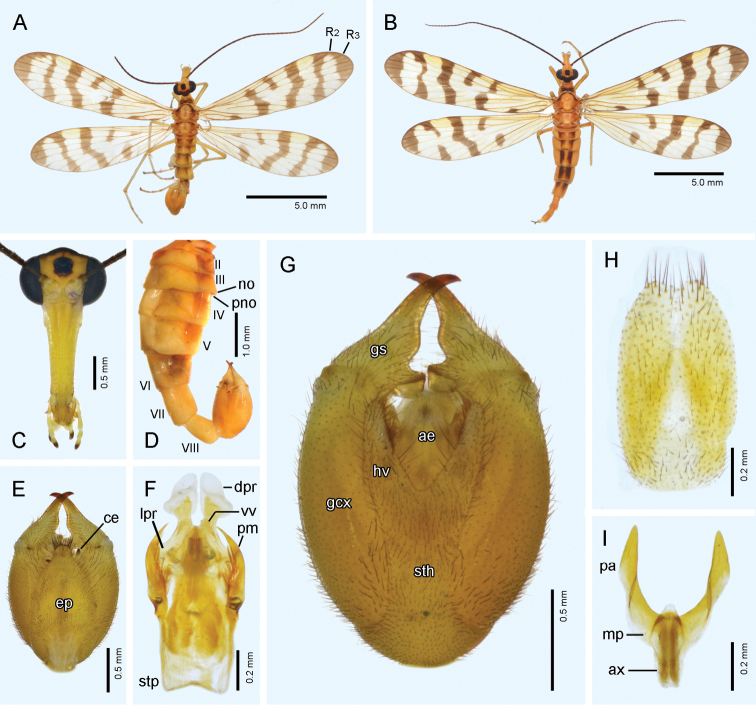
*Panorpa menqiuleii* sp. nov. **A, C–G** male **B, H, I** female. **A, B** Habitus, dorsal view **C** head, frontal view **D** abdomen, lateral view **E, G** genital bulb, dorsal and ventral views, respectively **F** aedeagal complex, ventral view **H** subgenital plate, ventral view **I** medigynium, ventral view. **ae**, aedeagus; **ax**, axis; **ce**, cercus; **dpr**, dorsal process; **ep**, epandrium; **gcx**, gonocoxite; **gs**, gonostylus; **hv**, hypovalve; **lpr**, lateral process; **mp**, main plate; **no**, notal organ; **pa**, posterior arm; **pm**, paramere; **pno**, postnotal organ; **sth**, stalk of hypandrium; **stp**, stalk of paramere; **vv**, ventral valve.

*Head* (Fig. 3C). Vertex and frons yellow. Transverse black pattern on occiput extending to border of compound eyes laterally, and connected to smaller black spot enclosing ocellar triangle through a thin black line anteriorly. Rostrum yellow, sparsely covered with short black setae, with its length approximately 2.6 times as long as basal width. Labrum yellowish brown. Maxillary palp with basal four segments and basal half of distal segment yellow, and apical half of distal segment black. Scape yellow, pedicel yellowish brown, flagellomeres 32‒34, mostly black but dark brown in basal two or three.

*Thorax* (Fig. 3A). Pronotum unevenly yellowish brown with two dark-brown transverse stripes, and bearing 10‒12 stout setae along anterior margin. Meso- and metanotum light yellowish brown mesally, brown laterally and dark brown at anterior margin, sparsely covered with short setae; scutellar arms slightly deepened. Pleura and legs light yellowish brown, with distal tarsomere blackish.

*Wings* (Fig. 3A). Membrane subtranslucent, slightly tinged with whitish yellow and fading toward apex. Markings blackish brown, dentate along longitudinal veins. Veins dark brown except apical crossveins pale. Pterostigma light yellow. Forewing apical band broad; pterostigmal band with apical branch detached and greatly elongated anteriorly, and connected with apical band along costal margin; basal branch bent inward; marginal spot extending from Sc to beyond R_4+5_; basal band complete or split into two large spots; basal spot shifted posteriorly along anal margin; R_2_ unfurcated. Hindwing similar to forewing but with relatively reduced markings; basal spot absent.

*Abdomen* (Fig. 3A, D). Terga II‒V yellow mesally and strongly darkened laterally, sparsely covered with black short setae; corresponding sterna light yellow. Notal organ on posterior margin of T3 slightly prolonged posteriorly with a rounded apex, and covering acute postnotal organ on anterior portion of T4. A6‒A8 yellow, cylindrical. A6 as long as A5, without anal horns. A7 slightly shorter and narrower than A6. A8 slightly shorter and narrower than A7, slightly enlarged posteriorly toward beveled apex.

Genital bulb (Fig. 3E, G) bold oval, mostly yellow except apex of gonostyli blackish. Epandrium broad, oval with abruptly narrowed apex bearing numerous long and dense setae. Cerci clavate, yellow in basal half and black in distal half. Hypandrium with long broad stalk and a pair of shorter hypovalves; each hypovalve slightly tapering toward apex, and bearing long stout setae along inner margin. Gonocoxites stout, approximately 1.6 times as long as gonostyli; gonostyli bearing a rounded median tooth and a large subtrapezoidal basal process on inner margin. Parameres (Fig. 3F) short, slightly curved inward, with greatly expanded stalk basally; not exceeding apex of ventral aedeagal valves; and bearing a row of long spines along inner margin. Ventral aedeagal valves simple and short; dorsal processes constricted neck-like basally and greatly enlarged apically; lateral processes short and stout.

#### Description-female.

Similar to males except relatively denser wing markings and darker terga (Fig. 3B).

*Female genitalia* (Fig. 3H, I). Subgenital plate long oval, with a shallow V-shaped terminal emargination, and bearing long stout setae marginally. Medigynium with poorly developed main plate; posterior arms long, twisted ventrally in distal half; axis shorter than posterior arms, with parallel apodemes extending slightly beyond main plate.

#### Distribution.

China, Anhui (Yuexi and Huoshan Counties).

#### Remarks.

Two male-unknown species, *Panorpa pusilla* Cheng, 1949 from Shaanxi and *Panorpa pieli* Cheng, 1957 from Jiangxi, are probably related to *P. menqiuleii* sp. nov. by the unbranched R_2_ in both fore- and hindwings, and the twisted posterior arms and short axis in female medigynium. The black pattern on the occiput, however, can readily distinguish *P. menqiuleii* sp. nov. from these two species.

### Panorpa mokansana

Taxon classificationAnimaliaMecopteraPanorpidae

Cheng, 1957

FFC53FFBEE3957D38A36F1AE7A7C1516

Figure 4

Panorpa mokansana Cheng, 1957a: 27, figs 1, 2.
Panorpa wrighti Cheng, 1957a: 28, figs 3, 4; P. wrightae nom. corr., Wang & Hua, 2017: 31. syn. nov.

#### Materials examined.

2♂17♀, CHINA: Zhejiang Province, Deqing County [德清县], Mount Mogan [莫干山], Weird Rock Corner [怪石角] (30°36'34"N, 119°50'58"E, 640 m), 8.x.2018, leg. Ji-Shen Wang.

#### Measurements.

Male FL 10.8‒12.0 mm, FW 2.9‒3.2 mm; HL 9.8‒10.7 mm, HW 2.8‒3.0 mm. Female FL 12.9‒13.5 mm, FW 3.3‒3.5 mm; HL 11.9‒12.5 mm, HW 3.0‒3.3 mm.

#### Redescription-male.

**Figure 4.**
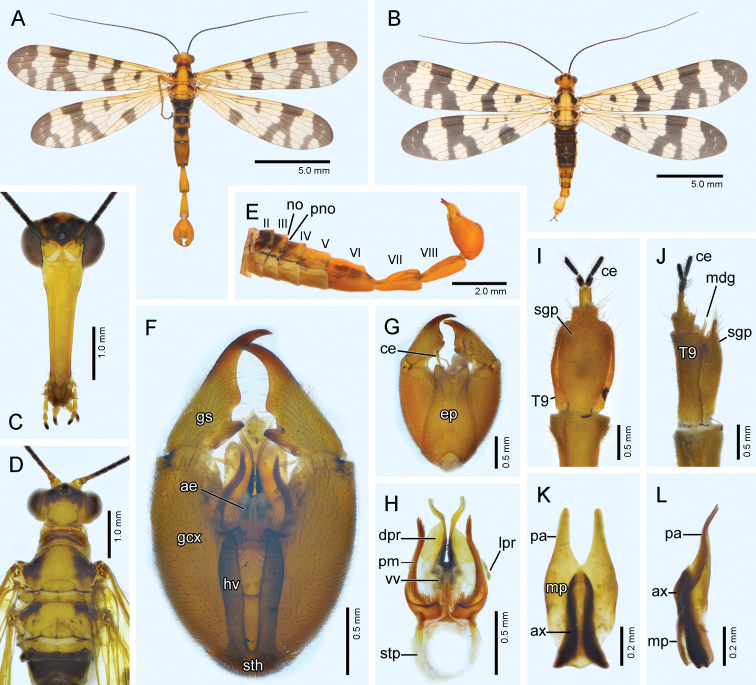
*Panorpa mokansana* Cheng, 1957 **A, C–H** Male **B, I–L** female. **A, B** Habitus, dorsal view **C** head, frontal view **D** dorsum, dorsal view **E** abdomen, lateral view **F, G** genital bulb, ventral and dorsal views, respectively **H** aedeagal complex, ventral view **I, J** A8‒A11, ventral and lateral views, respectively **K, L** medigynium, ventral and lateral views, respectively. **ae**, aedeagus; **ax**, axis; **ce**, cercus; **dpr**, dorsal process; **ep**, epandrium; **gcx**, gonocoxite; **gs**, gonostylus; **hv**, hypovalve; **lpr**, lateral process; **mdg**, medigynium; **mp**, main plate; **no**, notal organ; **pm**, paramere; **pno**, postnotal organ; **sgp**, subgenital plate; **sth**, stalk of hypandrium; **stp**, stalk of paramere; **vv**, ventral valve.

*Head* (Fig. 4A, C). Vertex yellow. Large black pattern enclosing ocelli, and extending posteriorly, forming thin black margin aside yellow occiput. Rostrum yellowish brown and deepened toward apex, with its length approximately 3.7 times as long as basal width. Maxillary palp with basal four segments yellowish brown and distal segment dark brown. Scape yellowish brown; pedicel dark brown; flagellum black with 39‒41 flagellomeres.

*Thorax* (Fig. 4A, D). Pronotum unevenly dark brown and bearing 8‒10 stout setae along anterior margin. Meso- and metanotum dark brown antero-laterally, with a broad yellow mesal stripe; scutellar arms dark brown; postnota yellow. Pleura and legs yellowish brown.

*Wings* (Fig. 4A). Membrane hyaline, slightly tinged with yellow and fading toward apex; markings black; veins yellowish brown except apical crossveins pale white; pterostigma indistinct. Forewing apical band broad, with a large hyaline window in posterior portion; pterostigmal band complete, with basal branch two times as wide as apical branch; marginal spot thick and nearly extending to anterior border of thyridium; basal band broad, with posterior half two times as wide as anterior half; basal spot large and irregular; R_2_ bifurcated. Hindwing similar to forewing, but marginal spot reduced and not reaching C anteriorly; basal band represented by a large spot along hind margin and an indistinct small spot along anterior margin; and lacking a basal spot.

*Abdomen* (Fig. 4A, E). T2‒T5 black anteriorly and reddish brown posteriorly; corresponding sterna reddish brown. Notal organ on T3 broad, very short, bearing numerous dense setae posteriorly, and covering acute postnotal organ on T4. A6‒A8 reddish brown. A6 with irregular black pattern on lateral surface, approximately two times as long as A5, subcylindrical, slightly tapering from middle toward abruptly beveled apex. A7 with sooty black texture on lateral surface, greatly constricted stalk-like basally, and greatly enlarged towards truncated apex. A8 similar to A7 but less constricted basally, and rounded apically.

Genital bulb (Fig. 4F, G) reddish brown, oval. Epandrium long and broad, with wide V-shaped emargination terminally and forming a pair of stout processes laterally. Cerci long clavate, yellowish brown with slightly deepened apex. Hypandrium with greatly reduced stalk and a pair of slender hypovalves extending to middle of gonocoxites, and each bearing a row of long setae on inner margin of apical half. Gonocoxites stout, bearing a few long setae on ventral apex. Gonostyli longer than half length of gonocoxites, with prominent middle tooth and stout basal process on inner margin. Paramere (Fig. 4H) bifurcated: ventral branch short and stout, curved mesally; dorsal branch long and slender; both branches bearing numerous long spines along posterior margin. Ventral aedeagal valves membranous and inconspicuous; dorsal process broad basally, slender and curved divergently at distal portion; lateral process stout and inconspicuous.

#### Description-female.

Similar to males but darker in body color and denser in wing markings (Fig. 4B). T2‒T5 dark brown but reddish brown on hind margins; T6‒T10 reddish brown. T9 elongated, nearly 1.5 times as long as and wider than T8 , with its lateral margins greatly curled ventrad and enclosing lateral margin of subgenital plate (Fig. 4I, J).

*Female genitalia*. Subgenital plate (Fig. 4I, J) long oval with narrow base, broadest at distal fourth, tapering into subtriangular and indistinctly emarginate apex. Medigynium (Fig. 4K, L) with well-developed main plate; a pair of posterior arms slender and parallel, shorter than main plate, and slightly bending dorsad at distal half; axis approximately as long as main plate, not exceeding main plate posteriorly but slightly exceeding the latter anteriorly; apodemes greatly divergent at shortly bifurcated apexes, with anterior portion covered by main plate ventrally.

#### Distribution.

China, Zhejiang: Deqing County (Mount Mogan).

#### Remarks.

*Panorpa mokansana* Cheng, 1957 and *Panorpa wrightae* Cheng, 1957 were described from the same locality, Mount Mogan, based on a single male (19.ix.1927) and a single female (28.ix.1927), respectively (Cheng 1957a). The holotypes of these two nominal species are deposited in the Collection of California Academy of Sciences, San Francisco, California. According to Cheng’s descriptions, the female of *P. wrightae* resembles the male of *P. mokansana* in wing markings but only differs from the latter in the body color (dull brown vs. mostly reddish brown). During our recent expedition in Zhejiang Province, a number of new materials were collected from the type locality. The males have been readily determined to be *P. mokansana*, and the females conform to Cheng’s description and illustration of *P. wrightae*. Because females are essential for an insect species, it is reasonable for us to consider that *P. mokansana* and *P. wrightae* are very likely conspecific. Therefore, *P. wrightae* is treated as a junior subjective synonym of *P. mokansana* herein.

### Key to males of species of *Panorpa* from eastern China

(Three species are not included because the males are unknown: *P. klapperichi* Tjeder, 1950, *P. implicata* Cheng, 1957 and *P. pieli* Cheng, 1957)

**Table:** 

1	A7 and A8 cylindrical, not constricted basally	**2**
–	A7 and A8 constricted basally and enlarged toward apex	**7**
2	A6–A8 much longer than preceding segments; gonostyli approximately as long as gonocoxites	**3**
–	A6–A8 shorter than or as long as preceding segments; gonostyli much shorter than gonocoxites	**4**
3	Gonostyli bearing three small protuberances on apical half of inner margin; basal stalk of hypandrium three times as long as hypovalves (Fujian)	***Panorpa kellogi* Cheng, 1957**
–	Gonostyli lacking protuberances on inner margin; hypandrium with extremely reduced basal stalk and split into a pair of hypovalves basally (Jiangsu)	***Panorpa baohwashana* Cheng, 1957**
4	Wing membrane hyaline; dorsum of body dark brown; paramere bifurcated (Jiangxi)	***Panorpa obliqua* Carpenter, 1945**
–	Wing membrane tinged with yellow; dorsum of body yellow to yellowish brown; paramere simple	**5**
5	R_2_ in both fore- and hindwings bifurcated; apex of epandrium broad and rounded (Zhejiang)	***Panorpa jinhuaensis* sp. nov.**
–	R_2_ in both fore- and hindwings simple; apex of epandrium abruptly narrowed	**6**
6	Occiput yellowish brown; each hypovalve with a small rounded process on basal portion of inner margin (Jiangxi)	***Panorpa waongkehzengi* Navás, 1935**
–	Occiput with a black pattern; hypovalve straight on inner margin (Anhui)	***Panorpa menqiuleii* sp. nov.**
7	T6 with an anal horn at apex	**8**
–	T6 lacking an anal horn	**9**
8	A7 stalk-like at base and abruptly enlarged toward apex; paramere bifurcated (Zhejiang)	***Panorpa anfracta* Ju & Zhou, 2003**
–	A7 evenly enlarged toward apex; paramere simple (Zhejiang, Fujian)	***Panorpa kiautai* Zhou & Wu in Zhou et al., 1993**
9	Pterostigmal band in both fore- and hindwings lacking an apical branch	**10**
–	Pterostigmal band in both fore- and hindwings with an apical branch	**13**
10	Gonostyli with a large concavity on basal half of ventral surface (Jiangxi)	***Panorpa cladocerca* Navás, 1935**
–	Gonostyli lacking a concavity on ventral surface	**11**
11	Gonocoxites with dense stout setae on inner margin (Fujian, Jiangxi)	***Panorpa trifasciata* Cheng, 1957**
–	Gonocoxites lacking dense stout setae on inner margin	**12**
12	Median tooth of gonostyli acute; apex of paramere bulbous (Zhejiang)	***Panorpa cheni* Cheng, 1957**
–	Median tooth of gonostyli rounded; paramere slender, sword-shaped (Zhejiang)	***Panorpa choui* Zhou & Wu in Zhou et al., 1993**
13	Paramere simple	**14**
–	Paramere bifurcated	**15**
14	Paramere greatly elongated and extending beyond middle of gonostyli (Fujian)	***Panorpa flavicorporis* Cheng, 1957**
–	Paramere short and not exceeding apex of gonocoxites (Fujian)	***Panorpa fukiensis* Tjeder, 1950**
15	Wing membrane strongly tinged with yellow; two branches of paramere approximately equal in length	**16**
–	Wing membrane hyaline or slightly tinged with yellow; two branches of paramere distinctly unequal in length	**17**
16	Genital bulb long oval; paramere slender, extending beyond apex of gonocoxites (Fujian, Jiangxi, Zhejiang)	***Panorpa aurea* Cheng, 1957**
–	Genital bulb broad oval; paramere short, not exceeding apex of gonocoxites (Anhui, Zhejiang)	***Panorpa lutea* Carpenter, 1945**
17	Paramere with ventral branch two-thirds as long as dorsal branch (Jiangxi)	***Panorpa coomani* Cheng, 1957**
–	Paramere with ventral branch shorter than half length of dorsal branch	**18**
18	Aedeagus with dorsal valves finger-like and parallel (Jiangxi, Zhejiang)	***Panorpa tetrazonia* Navás, 1935**
–	Aedeagus with dorsal valves slender and greatly diverged apically (Zhejiang)	***Panorpa mokansana* Cheng, 1957**

## Discussion

By adding two new species and synonymizing one species, the species number of *Panorpa* from eastern China is updated to 22.

Evidently, *Panorpa jinhuaensis* sp. nov., *Panorpa menqiuleii* sp. nov. and *Panorpa waongkehzengi* Navás, 1935 are more or less related to the northeastern Asiatic *Panorpa amurensis*, *Panorpa japonica* and *Panorpa kongosana* groups by the following characters: in males, 1) cylindrical A6‒A8; 2) long stalk of hypandrium; 3) greatly expanded stalk of paramere; and in females, 4) twisted posterior arms and weakly or moderately developed main plate in medigynium. In addition, basally crossed male parameres occur only in a small number of species in *Panorpa* (all six species in the *P. amurensis* group, ca. nine species out of eleven in the *P. japonica* group, all three species in the *P. kongosana* group, and *P. jinhuaensis* sp. nov.), likely suggesting their close affinities.

Bivoltinism is frequently reported in some species of *Panorpa*. For example, *Panorpa liui* Hua, 1997 from the *Panorpa amurensis* group (Jiang and Hua 2013), *Panorpa japonica* Thunberg, 1784 from the *Panorpa japonica* group (Ogai 1999), *Panorpa qinlingensis* Chou & Ran in Chou et al., 1981 from central China (Cai and Hua 2009), *Panorpa communis* Linnaeus, 1758, *Panorpa nigrirostris* MacLachlan, 1882 and *Panorpa vulgaris* Imhoff & Labram, 1845 from the *P. communis* group (Sauer et al. 2003; Vermeulen et al. 2009; Dvořák and Ghahari 2016), and presumably, *Panorpa nuptialis* Gerstaecker, 1863 from North America (Byers 1963).

*Panorpa jinhuaensis* sp. nov. is likely a bivoltine insect species. The spring generation (Fig. 1B) was observed to fly in late April, and the summer generation (Fig. 1C‒E) in early October. Most species of *Panorpa* prefer cool habitats, and often inhabit high-elevated mountainous regions in the subtropical zone, especially in southern and eastern China (Wang and Hua 2016, 2017; Hua et al. 2018). In the low elevated habitat (ca. 100 m a.s.l) of *P. jinhuaensis* sp. nov., however, bivoltinism may give the insect an advantage to avoid the hot summer from June to August (29‒33 °C in Jinhua City), and thus enables them to breed in the lowlands in the cooler spring and autumn months. Further investigations are needed to reveal its life history.

## Supplementary Material

XML Treatment for Panorpa jinhuaensis

XML Treatment for Panorpa menqiuleii

XML Treatment for Panorpa mokansana
